# Cardiac Abnormalities in a Predictive Mouse Model of Chagas Disease

**DOI:** 10.3390/pathogens12111364

**Published:** 2023-11-17

**Authors:** Amanda Fortes Francisco, Giovane R. Sousa, Mhairi Vaughan, Harry Langston, Archie Khan, Shiromani Jayawardhana, Martin C. Taylor, Michael D. Lewis, John M. Kelly

**Affiliations:** 1 Department of Infection Biology, London School of Hygiene and Tropical Medicine, London WC1E 7HT, UK; 2 Harvard Medical School, Section on Immunobiology, Joslin Diabetes Center, 1 Joslin Place, Boston, MA 02215, USA; 3 Research Department of Haematology, Cancer Institute, Faculty of Medical Sciences, University College London, London WC1E 6DD, UK

**Keywords:** *Trypanosoma cruzi*, electrocardiography (ECG), chronic Chagas cardiomyopathy, Chagas heart disease

## Abstract

Chronic Chagas cardiomyopathy (CCC) results from infection with the protozoan parasite *Trypanosoma cruzi* and is a prevalent cause of heart disease in endemic countries. We previously found that cardiac fibrosis can vary widely in C3H/HeN mice chronically infected with *T. cruzi* JR strain, mirroring the spectrum of heart disease in humans. In this study, we examined functional cardiac abnormalities in this host:parasite combination to determine its potential as an experimental model for CCC. We utilised electrocardiography (ECG) to monitor *T. cruzi*-infected mice and determine whether ECG markers could be correlated with cardiac function abnormalities. We found that the C3H/HeN:JR combination frequently displayed early onset CCC indicators, such as sinus bradycardia and right bundle branch block, as well as prolonged PQ, PR, RR, ST, and QT intervals in the acute stage. Our model exhibited high levels of cardiac inflammation and enhanced iNOS expression in the acute stage, but denervation did not appear to have a role in pathology. These results demonstrate the potential of the C3H/HeN:JR host:parasite combination as a model for CCC that could be used for screening new compounds targeted at cardiac remodelling and for examining the potential of antiparasitic drugs to prevent or alleviate CCC development and progression.

## 1. Introduction

Chagas disease (CD) is caused by the insect-transmitted protozoan parasite *Trypanosoma cruzi* and has a major impact on public health throughout Latin America, where six to seven million people are infected [1]. Due to migration, CD is increasingly being diagnosed in non-endemic regions, particularly the US and Europe. Globally, infection with *T. cruzi* is the most common cause of infectious cardiomyopathy [2]. The initial acute stage of the disease is characterised by a widely disseminated infection and a readily detectable parasitaemia. In most cases, symptoms are mild and nonspecific, although, on occasions, there can be serious and life-threatening consequences, including myocarditis or meningoencephalitis [3,4]. After 2–8 weeks, with the induction of an adaptive immune response, in which CD8^+^ IFN-γ^+^ T cells play a major role, the infection typically transitions to an asymptomatic chronic stage in which the parasite burden is extremely low [5,6]. Approximately 30% of infected individuals will develop chronic Chagas cardiomyopathy (CCC), although the condition can take many years or decades to become symptomatic. This can give rise to a wide spectrum of manifestations, ranging from minor myocardial involvement to left ventricular systolic dysfunction, dilated cardiomyopathy (myofiber hypertrophy and interstitial fibrosis), arrhythmias, thromboembolic events, and terminal cardiac failure, including sudden death [3,7,8]. Electrocardiography (ECG) has played a key role in CD patient evaluation and management [9,10]. It represents a low-cost diagnostic method and can be predictive of CCC outcomes [11,12].

Overall, individuals with CD have a 40% prevalence of ECG abnormalities [9], and conditions such as right bundle branch block (RBBB) can be up to 15 times higher than in seronegative individuals [13]. In children and young adults, the incidence of ECG alterations has been found to be similar to that in older adults, suggesting a potential for early onset cardiac disease [9,13]. Identifying the triggers of ECG changes and their relative importance has been difficult in humans because of the long period over which CCC develops and the highly diverse nature of the symptoms. The mechanisms that drive these cardiac abnormalities in CD patients have been the subject of multiple studies focused on the central question of whether parasite persistence is required to cause those changes. Experimental studies have shown that cardiac disease intensity can be linked to infection with specific parasite strains [14,15], and that such outcomes are also influenced by the genetic background of the animal model [16,17,18]. However, the role of other factors, such as environment, nutrition, and health status, and how they contribute to the effectiveness of the host immune response and the development, or otherwise, of CCC are less well understood.

There is now a general view that the presence of the parasite is required to promote the development of CCC [18,19,20]. However, in experimental murine models of CD, cardiac damage is often present in the absence of locally detectable infection at the time of tissue sampling [16,21]. This has led to the suggestion that transient episodes of low-level *T. cruzi* infection of the heart trigger cycles of inflammation that result in local clearance of parasites but give rise to cumulative damage to the myocardium [22]. Elucidating the underlying mechanisms responsible for myocardial dysfunction in CD has major implications for diagnosis, risk stratification, and clinical management. This highlights the need for improved predictive models to aid research on disease pathogenesis and therapies that might reverse or block the resulting pathology. It is a requirement of such models that they capture the heterogeneous nature of human Chagas heart disease in terms of both the temporal profile and disease severity.

Here, we evaluated C3H/HeN mice infected with *T. cruzi* JR strain as an experimental model to monitor the development of heart disease. In terms of cardiac fibrosis, this infection model gives rise to a spectrum of pathology ranging from modest to severe [16,18]. Using highly sensitive in vivo imaging technology, histological analysis, and non-invasive, anaesthesia-free ECG, we undertook a longitudinal analysis of Chagas heart disease development during acute and chronic infections. This has allowed us to monitor the progressive impact of *T. cruzi* infection on several parameters of cardiac function relevant to human CD.

## 2. Materials and Methods

### 2.1. Mice and Parasites

Animal infections were performed under UK Home Office project licences PPL 70/8207 and PPL P9AEE04E4 and approved by the London School of Hygiene and Tropical Medicine Animal Welfare and Ethical Review Board (AWERB). All protocols and procedures were conducted in accordance with the UK Animals (Scientific Procedures) Act 1986. Female BALB/c and C3H/HeN mice were purchased from Charles River (UK), and CB17 SCID mice were bred in-house. Animals were maintained under specific pathogen-free conditions in individually ventilated cages. They experienced a 12 h light/dark cycle, with access to food and water ad libitum. SCID mice were infected with 1 × 10^4^ culture trypomastigotes in 0.2 mL PBS via i.p. injection. Female C3H/HeN and BALB/c mice, aged 7–8 weeks, were infected by i.p injection with 1 × 10^3^ trypomastigotes derived from SCID mouse blood [23]. At experimental endpoints, mice were sacrificed by exsanguination under terminal anaesthesia. To reflect the genetic diversity of *T. cruzi*, we selected strains JR (discrete typing unit (DTU) I) and CL Brener (DTU VI), both of which were modified to express a red-shifted luciferase [16,21,24]. Initially, 3 infection combinations were investigated based on previous studies which had revealed a range of cardiac fibrosis outcomes [16,18]: strain JR in C3H/HeN mice, strain CL Brener in C3H/HeN mice, and strain CL Brener in BALB/c mice.

### 2.2. ECG Recording and Analysis

ECGenie (Mouse Specifics Inc., Boston, MA, USA) is a rapid non-invasive device that facilitates ECG data acquisition from conscious mice without surgery, implants, or anaesthesia [25,26,27]. The instrument detects cardiac electrical activity through direct contact between the mouse paws and disposable footplate electrodes. For data recording, mice were gently removed from their cages and positioned on the ECG platform. An array of gel-coated ECG electrodes embedded in the floor of the platform provided contact with paws. To avoid handling-induced alterations in heart rate, each mouse was permitted to acclimatise on the platform for at least 5 min prior to collection of baseline data. An average of ~229 signals per mouse per time point were acquired to evaluate changes in the heart physiology during long-term *T. cruzi* infection.

Each signal was analysed using the software EzCG e-MOUSE, an internet-based physiological waveform analysis portal. The software incorporates Fourier analyses and linear time-invariant digital filtering of frequencies below 2 Hz and above 100 Hz to minimise environmental signal disturbances [25,26,27]. The software uses peak detection algorithms to detect R waves and a series of artificial intelligence algorithms to identify the PQRST morphology and interval durations. We cannot therefore comment on the amplitude of the ECG descriptors, though only signals in which clear PQRST morphology could be detected were included. After indeterminate signals were manually excluded, unbiased signal processing algorithms computed all ECG interval durations. Upon analysis, data were automatically compiled into a Microsoft Excel spreadsheet. Arrhythmias were identified by analyses of ECG tracings as irregular RR intervals (the time between R-waves of the QRS signal), premature atrial, or ventricular complexes as well as the presence of broad complex tachycardia.

### 2.3. In Vivo and Ex Vivo Bioluminescence Imaging

For in vivo bioluminescence imaging, infected mice were injected with 150 mg kg^−1^ d-luciferin i.p., anaesthetised using 2.5% (*v*/*v*) isoflurane in oxygen for 2–3 min, and then imaged using an IVIS Spectrum system (Revvity, Hopkinton, MA, USA) [23]. Exposure times varied from 10 s to 5 min, depending on signal intensity. The detection threshold was established from uninfected mice. After imaging, mice were revived and returned to cages. To avoid any influence of isoflurane on cardiac data, we allowed a gap of at least 48 h between imaging and ECG analysis and minimised the total number of imaging events.

For ex vivo imaging, mice were injected with 150 mg kg^−1^ d-luciferin i.p. 5–7 min before terminal euthanasia using dolethal (200 mg kg^−1^). Trans-cardiac perfusion was performed with 10 mL of 0.3 mg mL^−1^ d-luciferin in DPBS (Dulbecco′s Phosphate Buffered Saline). Tissues and organs of interest were collected, and the heart was bisected along the coronal plane. Samples were soaked in DPBS containing 0.3 mg mL^−1^ d-luciferin. Bioluminescence imaging was performed as above. To estimate parasite burden in the heart tissue, regions of interest (ROIs) were drawn using Living Image 4.7.3 to quantify bioluminescence expressed as total flux (photons/second; p/s). Data from infected mice were normalised using matching hearts from uninfected mice to establish the fold change in radiance [21].

### 2.4. Cardiac Histopathological Analysis

For in-depth assessment of cardiac physiological changes during *T. cruzi* infection, mice were euthanised using dolethal (200 mg kg^−1^), and heart tissue samples were collected. They were fixed in either 10% neutral buffered formalin (Sigma, St. Louis, MO, USA) or Glyo-Fixx (Epredia, Kalamazoo, MI, USA) then dehydrated in ethanol, cleared in Histo-clear, and embedded in paraffin. Three-micron heart sections were stained with haematoxylin and eosin (H&E) and analysed employing a DFC295 camera attached to a DM3000 light-emitting diode microscope (Leica, Wetzlar, Germany) using an ocular lens with 20× magnification. Images were digitalised for histomorphometric analysis using the software Leica Application Suite V.4.12. For the inflammatory index, the total number of cells were counted in 10 microscope fields (area 2.66 × 10^5^ μm^2^) from across the base of the heart (5 fields from different sides of the heart) at 200× total magnification per tissue section. The inflammation index for each animal was calculated as the average of the 10 randomly selected images. Each individual mouse received a code so that tissue section analysis could be blinded [18].

### 2.5. Immunohistochemistry for iNOS Detection

For antigen retrieval, deparaffinised heart sections were submerged in 10 mM sodium citrate buffer (pH 6.0) containing 0.05% Tween-20 and heated to 96 °C for 30 min. Sections were then blocked in 2.5% normal horse serum. Endogenous peroxidase activity was quenched by incubating with 3% hydrogen peroxide. Slides were then incubated with rabbit polyclonal anti-iNOS IgG antibody (ab15325, Abcam, Cambridge, UK) at a 1:50 dilution at 4 °C overnight. The secondary antibody ImmPRESS horse anti-rabbit IgG peroxidase polymer (VectorLabs) was used at its stock concentration without dilution. The reaction was visualised by incubating the section with 3,3′-diaminobenzidine tetrahydrochloride ImmPACTTM DAB substrate kit (VectorLabs) [28]. Images were digitalised for histomorphometric analysis using the software Leica Application Suite V.4.12. For iNOS analysis, 15 images were taken across the whole heart, 5 of which were from the base (ventricular area). The top of the heart consists primarily of the atria and the proximal part of the coronary vessels; adipose-rich areas were also found in some tissues. The number of DAB+ (brown) pixels was counted in 15 microscope fields (area 2.66 × 10^5^ μm^2^) at 200× total magnification per tissue section. The iNOS index was quantified as the total DAB+ area per image for each animal and calculated as the average of the 15 randomly selected images. As above, analysis of tissue sections was carried out under blinded conditions.

### 2.6. RT-qPCR

Heart tissue samples were snap frozen on dry ice and stored at −70 °C. For RNA extraction, samples were thawed and homogenised in 1 mL Trizol (Invitrogen, Carlsbad, CA, USA) per 30–50 mg of tissue using a Precellys 24 homogeniser (Bertin). To each sample, 200 µL of chloroform was added and mixed by vortex, after which the phases were separated by centrifugation at 13,000× *g* at 4 °C. RNA was extracted from the aqueous phase using the RNeasy Mini Kit (Qiagen, Venlo, The Netherlands) with on-column DNAse digestion, as per manufacturer’s protocol. The quantity and quality of RNA were assessed using Qubit Fluorimeter (Thermofisher, Waltham, MA, USA). cDNA was synthesised from 1 µg of total RNA using Superscript IV VILO mastermix (Invitrogen), as per manufacturer’s protocol, in reaction volumes of 20 μL. qPCR reactions contained 4 µL of cDNA (1:50 dilution) and 100 nM of each primer (Table 1) and QuantiTect SYBR Green PCR master mix (Qiagen). Reactions were run in duplicate using an Applied Biosystems 7500 fast RT-PCR machine (Thermofisher) using reaction conditions of 15 min at 95 °C (holding) followed by 40 cycles of 15 s at 94 °C, 30 s at 60 °C, and 30 s at 72 °C. Each plate included a duplicate no-template control. Data were analysed by the ΔΔCt method [29] using murine Gapdh as the endogenous control gene. The significance level for the fold difference in gene expression in the infected group was set at the average fold difference ± 2 standard deviations (SDs) of the non-infected group.

### 2.7. Statistical Analyses

Individual animals were used as the unit of analysis unless otherwise stated. Groups were compared with the Kruskal–Wallis test for continuous variables and the chi-square test for categorical variables, and corrected for multiple testing with the post hoc Dunnett’s test or the Benjamini–Hochberg procedure to control the false discovery rate at 5%. The statistical analysis was conducted with GraphPad Prism 10.0.3 and SAS 9.4. For the histopathological investigation, unpaired two-sided Student’s *t*-tests were performed. A value of *p* < 0.05 was considered significant.

## 3. Results

### 3.1. Longitudinal ECG Monitoring of T. cruzi-Infected Mice

**Figure 1:**
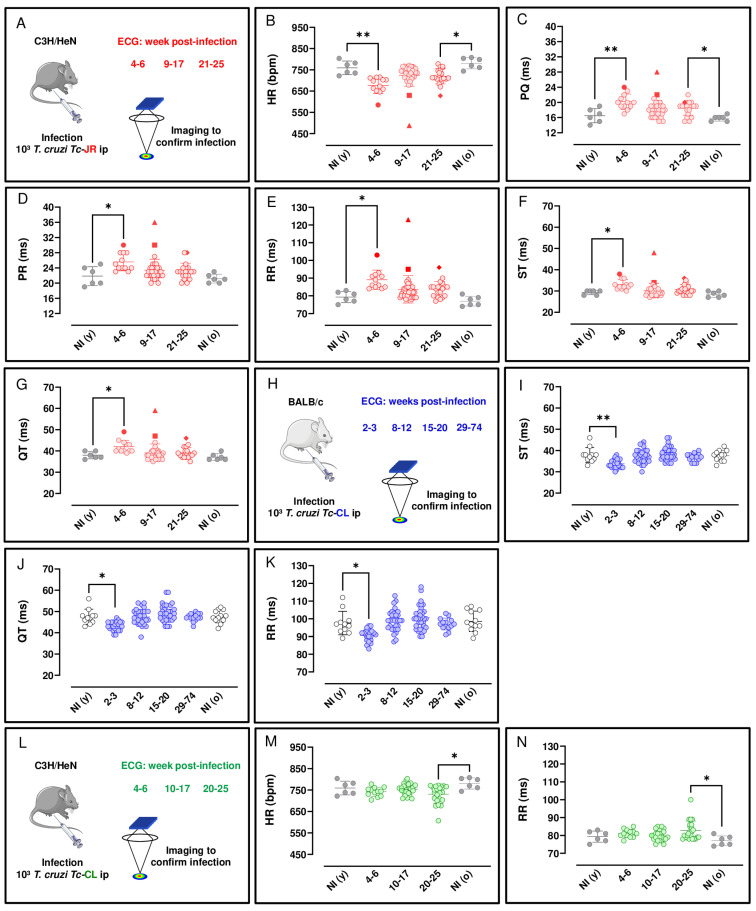
Longitudinal ECG analysis of C3H/HeN and BALB/c mice during the course of infection with *T. cruzi* strains JR and CL. (**A**,**H**,**L**) Outline of monitoring strategy (parts of the figure were drawn by using pictures from Servier Medical Art; Servier Medical Art by Servier is licensed under a Creative Commons Attribution 3.0 Unported License (https://creativecommons.org/licenses/by/3.0/, accessed on 15 September 2022). Parameters measured (**B**–**G**) to monitor C3H/HeN at the acute (4–6 wpi, n = 12) and chronic stages (9–17 wpi, n = 35; 21–25 wpi, n = 20) of *T. cruzi* infection. Parameters measured (**I**–**K**) to monitor BALB/c infections during the acute (2–3 wpi, n = 25) and chronic stages (8–12 wpi, n = 32; 15–20 wpi, n = 33; 29–74 wpi, n = 14) of infection. Age-matched BALB/c controls consisted of non-infected young NI (y), n = 12 and non-infected old NI (o), n = 12 mice which were assessed at the beginning and end of the experiment, respectively. Parameters measured (**M**,**N**) to monitor C3H/HeN infections during the acute (4–6 wpi, n = 12) and chronic stages (10–17 wpi, n = 24; 20–25 wpi, n = 23) of infection. Age-matched C3H/HeN controls consisted of NI (y), n = 6 and NI (o), n = 6 mice which were assessed at the beginning and end of the experiment, respectively. Each mouse analysed is represented by a dot. For all datasets, significance was determined using a Kruskal–Wallis test with Dunn’s post hoc correction. * *p* < 0.05, ** *p* < 0.001. Filled symbols (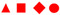) identify individual mice where some of the parameter values lie outside the typical range.

Several reports have described the use of ECG to assess cardiac function during experimental *T. cruzi* infection [30,31,32,33]. However, these systems have required anaesthesia, surgery, or implanted electrodes, interventions that can suppress cardiovascular activity and/or perturb heart rate (HR). As a more relevant approach, we used ECGenie, a cardiac monitoring device that allows rapid acquisition of data in a non-invasive, anaesthesia-free manner (Materials and Methods). First, we examined C3H/HeN mice infected with the *T. cruzi* JR strain, since severe cardiac fibrosis can develop in this infection model [18]. Longitudinal ECG monitoring was carried out during the acute stage, 4–6 weeks post-infection (wpi) in this model, and at various points following transition to the chronic infection (8 wpi onwards) [16]. There were no significant age-matched differences between non-infected mice assessed at the beginning and end of the experimental period (i.e., young vs. old). Significant ECG changes were observed as a result of infection. These included a reduction in HR during both acute (*p* = 0.001) and chronic (*p* = 0.014) stages of infection, relative to non-infected age-matched controls (Figure 1B). In line with the overall bradycardia, we observed prolonged PQ intervals (the period of atrial contraction) during both the acute (*p* = 0.009) and chronic (*p* = 0.025) stages (Figure 1C) relative to non-infected age-matched controls. Similarly, there was an increase in the PR interval during the acute stage relative to non-infected age-matched controls (*p* = 0.039) (Figure 1D). With the RR interval (the time between consecutive heart beats) and ST and QT intervals (two overlapping parameters that are indicators of ventricular repolarisation and depolarisation), there was an increase during the acute stage (*p* = 0.001, 0.019, and 0.044, respectively) relative to non-infected age-matched controls (Figure 1E–G). Therefore, acute and chronic infections of C3H/HeN mice with the *T. cruzi* JR strain were both associated with a greater prevalence of ECG conduction abnormalities. Specifically, there was a clear bradycardia phenotype, which was more pronounced in the acute phase, linked with increased interval times across the cardiac cycle. During the chronic phase, bradycardia persisted, but the association became more specifically linked to an increased PQ interval. No other changes were detected during acute or chronic stage infections in comparison with age-matched controls.

ECG correlations were observed in the C3H/HeN:JR mice during the acute stage (4–6 wpi) on infection. We found a significant, strong, negative association between HR x RR and QT, r(10) = −0.99 and −0.89, respectively, *p* < 0.001. A significant, strong, positive correlation was observed between PR x PQ, r(10) = 0.92, *p* < 0.001 and QT x ST and RR, r(10) = 0.92 and 0.90, respectively, *p* < 0.001.

Various ECG abnormalities were identified in this mouse model over the course of the infection with *T. cruzi*. During the acute stage, sinus bradycardia (SB) was detected in 33% of mice (4/12). This arrhythmia is defined by the presence of frequent sinus pauses in between bursts of normal activity, where a slower pulse rate originates from functional impairment of the heart sinus. In addition, during the chronic stage of infection, 9% of mice (3/38) displayed left anterior fascicular block (LAFB), an abnormality defined by axis left deviation and which may be associated with myocardial fibrosis. Thus, heart conduction disorders have a heterogeneous pattern in this infection model, with a range of symptoms that parallel the diverse nature of the pathology in human disease. Right bundle branch block (RBBB), a heart conduction disorder defined as the presence of broad, notched, or split QRS complexes, was also prevalent and was identified in 24% of mice (7/29) during the chronic stage. Also, ventricular extrasystole (VE), an arrhythmia that results in an extra beat due to premature ventricular contraction, was observed in one third of the acutely infected mice examined, although this fell to 15% (4/26) during the chronic stage of infection.

For comparison, we next examined BALB/c mice infected with the *T. cruzi* CL Brener strain on cardiac function. At the peak of the acute stage (2–3 wpi in this experimental model), we found that there was an decrease for ST, QT, and RR intervals at 2–3 wpi relative to non-infected age-matched controls (*p* = 0.006, 0.011 and 0.037, respectively) (Figure 1I, Figure 1J, and Figure 1K, respectively). No other changes were detected in this model during acute or chronic stage infections in comparison with age-matched controls.

We also investigated the cardiac impact of a C3H/HeN mice model infected with the *T. cruzi* CL Brener strain. In the acute stage, 4–6 wpi, there was no observable impact on HR, although by the chronic stage of infection (20–25 wpi), compared to non-infected age-matched controls, there was a significant reduction (*p* = 0.037) at a population level (Figure 1M). We also observed prolonged RR intervals, the mean duration between depolarisation repolarisation cycles (*p* = 0.032) during the chronic stage (Figure 1N) relative to non-infected age-matched controls. A predictable direct inverse relation between HR and RR interval duration was observed in the C3H/HeN mice infected with both parasite strains in this study (Figure 1B,E,M,N). No other changes in the measured ECG parameters were detected in these infected mice.

### 3.2. Analysis of Heart-Specific Infection

**Figure 2:**
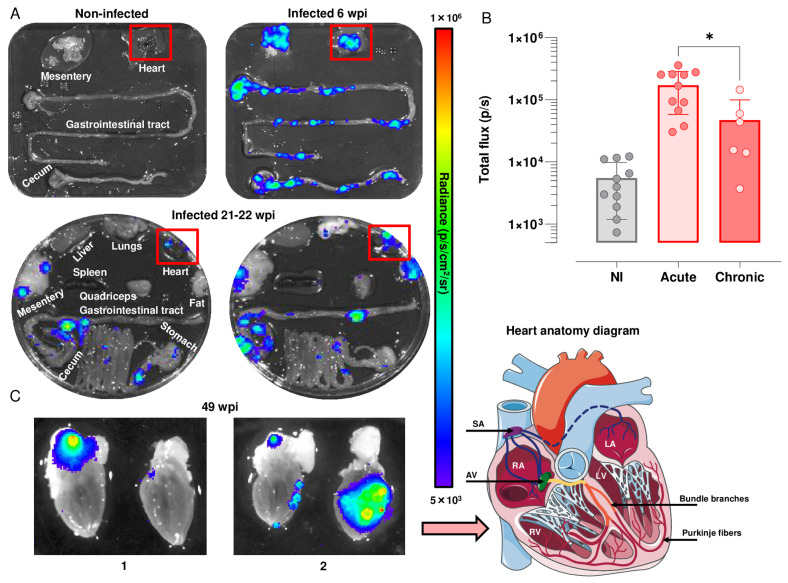
Cardiac parasite load in C3H/HeN mice during acute and chronic infection with *T. cruzi* strain JR. (**A**) Representative ex vivo bioluminescence images of mouse organs and tissues during acute (6 wpi) and chronic infection (21–22 wpi) with *T. cruzi* strain JR. The heat map is on a log10 scale and indicates the intensity of bioluminescence from low (blue) to high (red). (**B**) Quantification of cardiac parasite burden (red boxes in A indicate regions of interest containing the heart) in non-infected (NI, n = 11) and infected mice (acute stage, n = 11; chronic stage, n = 6) inferred from total flux (p/s). (**C**) Localisation of infection in bisected mouse heart sections that reveal the interior chambers in the late stage of chronic infection (1 and 2; 49 wpi). SA: sinoatrial node, AV: atrioventricular node, RA: right atrium, LA: left atrium, RV: right ventricle, LV: left ventricle (Figure modified with text and annotation after adaptation of “Heart (5)” from Servier Medical Art by Servier, licensed under a Creative Commons Attribution 3.0 Unported License). For dataset B, significance was determined using a Kruskal–Wallis test with Dunn’s post hoc correction. * *p* < 0.05.

We used highly sensitive bioluminescence imaging to further analyse temporal and spatial cardiac infection in the C3H/HeN:JR model. During the acute stage, *T. cruzi* is pan-tropic and parasites can be routinely detected in heart tissue by ex vivo imaging [16,21] (Figure 2A). The highly dynamic nature of the infection was reflected in a wide variation in the cardiac parasite load, with a mean total flux of 150,000 p/s, which was derived from values in individual mice varying from 50,000 to 360,000 p/s. There is a linear relationship between bioluminescence and parasite load in this range [21]. In the chronic stage, in line with previous reports [16], ~80% of hearts displayed bioluminescence positivity in this infection model, although the parasite burden was significantly lower (p/s) (*p* = 0.024) than in the acute stage, and again, there was considerable variability between individual mice (Figure 2A,B). This compares to a ~10% level when snapshots of cardiac infection were assessed during the chronic stage in BALB/c mice, a strain that is better able to restrict the parasites to more immunotolerant sites, such as the GI tract [16].

Mouse hearts were bisected into anterior and posterior halves to reveal all four chambers and cardiac valves. In the acute stage (6 wpi, n = 11), ex vivo image macroscopic analysis using Living Image 4.7.3 revealed intense bioluminescence over the entire cardiac muscle area and the aortic arch in both halves of the bisected heart in 55% of mice (n = 6) (Figure 2A). In the remaining animals (n = 5), widespread parasite distribution was restricted to one half of the bisected heart and was observed intermittently in the aortic arches. During the chronic stage (21–22 wpi, n = 6), parasites were more often localised in the epicardial adipose tissue, with only one mouse heart in this group being bioluminescence-negative (Figure 2A,B). In later stages of infection (49 wpi) (n = 3), parasite distribution was comparable to that at 21–22 weeks (Figure 2C), localised to the epicardial adipose, and close to the aorta and to both the left and right cranial vena cava. In other instances, additional bioluminescence was observed in the left and right ventricle (Figure 2C). Therefore, across the chronic stage of infection, the cardiac parasite burden is much reduced and more local than during the acute stage (Figure 2C).

### 3.3. Upregulation of Cardiac iNOS Expression during Acute and Chronic T. cruzi Infection

**Figure 3:**
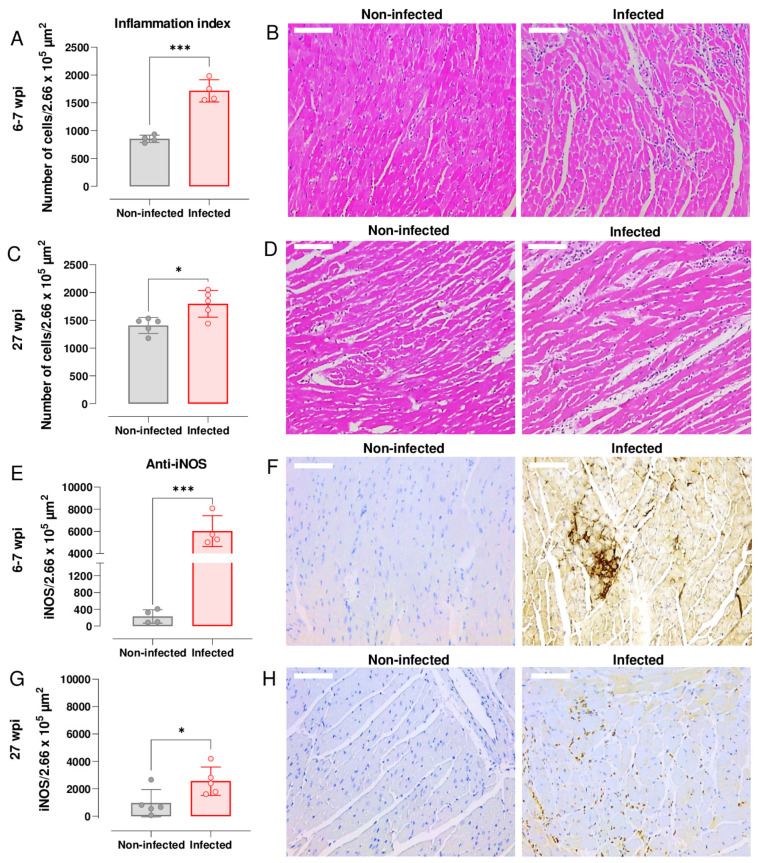
Cardiac inflammation and iNOS expression during acute and chronic infection of C3H/HeN mice with *T. cruzi* JR strain. (**A**) Quantitative histopathological analysis of cardiac tissue (number of nuclei per 2.66 × 10^5^ μm^2^) from non-infected mice and mice in the acute stage of infection (6–7 wpi, n = 4). (**B**) Inflammation indices were determined from H&E-stained heart photomicrographs (Materials and Methods). (**C**) Comparison of inflammation indices in cardiac tissue from non-infected mice and mice in the chronic stage of infection (27 wpi, n = 5). (**D**) H&E-stained heart photomicrographs from non-infected and chronically infected mice. (**E**) Quantitative anti-iNOS staining of cardiac tissue from non-infected mice and mice in the acute stage of infection (n = 4). Values were calculated from the brown pixels in tissue sections of 2.66 × 10^5^ μm^2^ as described (Materials and Methods). (**F**) Heart photomicrographs illustrating anti-iNOS staining (light and dark brown) during the acute stage of infection. Typically, cardiac photomicrographs derived from infected mice displayed a brown background due to the high level of iNOS. (**G**) Comparison of anti-iNOS staining in cardiac tissue from non-infected mice and mice in the chronic stage of infection (n = 5). (**H**) Anti-iNOS staining during the chronic stage of infection. In (**B**,**D**,**F**,**H**), white scale bars = 100 µm, magnification 200×. For all graphs, significance was determined using an unpaired *t*-test. * *p* < 0.05; *** *p* ≥ 0.0001.

Nitric oxide (NO) has a central role in controlling murine *T. cruzi* infections [34]. However, high levels of this free radical have also been linked with chronic cardiac pathology [35]. To investigate the temporal link between expression of iNOS and myocarditis in the C3H/HeN:JR infection model, we stained cardiac tissue sections with H&E and undertook histomorphometric analysis of digitalised images (Leica Application Suite V.4.12.; Materials and Methods). During the acute stage (6–7 wpi), the inflammation index was significantly greater in cardiac tissue from infected mice than from age-matched non-infected controls (*p* = 0.0002) (Figure 3A). Inflammatory infiltrates were widespread and were comprised predominantly of mononuclear cells with lymphocytic morphology. In some instances, the intensity of the cellular infiltrates forced the cardiac myofilaments to rearrange and adapt to a new tissue architecture (Figure 3B). Although cardiac inflammation continued at a similar level during the chronic stage (27 wpi), the differential intensity compared to non-infected tissue was less pronounced (*p* = 0.015; Figure 3C,D), mostly due to a higher baseline inflammation level in the age-matched control mice.

Enhanced expression of iNOS in response to infection is a feature of many types of inflammatory cells. To determine if the inflammatory infiltrates accounted for the greatly increased abundance of the iNOS, we immuno-stained cardiac tissue sections with anti-iNOS antibody (Materials and Methods). Compared to non-infected controls, the amount of staining was significantly higher in tissue from both acutely (*p* = 0.0002; Figure 3E,F) and chronically infected (*p* = 0.038; Figure 3G,H) mice. Staining was more intense during the acute stage and was distributed across the whole heart, although in the infected mice there was no correlation between the extent of cardiac inflammation, iNOS levels, or parasite burden. However, in the chronic phase (27 wpi), we found a significant negative association between myocarditis and iNOS levels, *r*(2) = −0.98, *p* = 0.001, perhaps reflecting differences in the ratio of inflammatory/anti-inflammatory immune cells within the cardiac infiltrate. Previously, we have shown that there is no significant association between endpoint myocarditis intensity and cardiac parasite loads during chronic infections in the C3H/HeN model [18].

### 3.4. Expression of Neuronal-Specific Genes in Cardiac Tissue during T. cruzi Infection

Immune-mediated neuronal damage has been postulated as a possible mechanism of pathogenesis in Chagas heart disease [2,36]. To gain insight into potential disruption of cardiac autonomic function in the C3H/HeN:JR host:parasite infection model, we assessed the expression of nine nervous system-related genes at 6 wpi (the late acute stage in this model) in addition to the inducible and endothelial nitric oxide synthases (Nos2/iNOS, Nos3/eNOS). When we examined the non-infected age-matched control group (six biological replicates in each case), there was variability in the level of gene expression. The vesicular acetylcholine transporter (VAChT) RNA showed the greatest variability (SD = 0.89), whilst the level of the neuronal nitric oxide synthase (nNOS) transcript, which has a role in protection against cardiac arrhythmia [37,38], was the most consistent between individual mice (SD = 0.10). Given this intrinsic variability, gene expression differences in infected mice were therefore only deemed significant if they were outside ±2 SDs of the non-infected controls.

**Figure 4:**
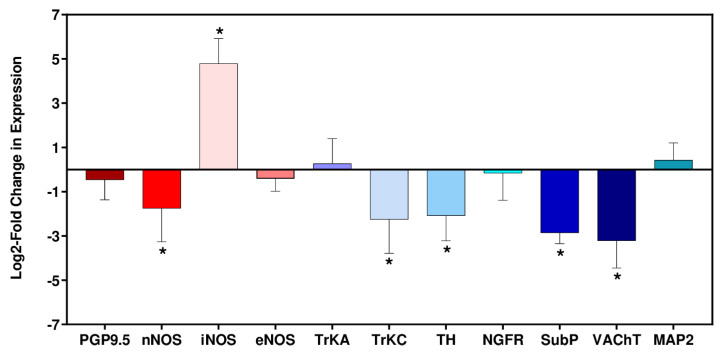
Log2 -fold change in RNA expression in cardiac tissue from C3H/HeN mice during acute stage infection with *T. cruzi* JR strain. Heart tissue was collected from mice 6 wpi (n = 6) and from age-matched non-infected mice (n = 6). RNA levels were determined by RT-PCR (Materials and Methods). Error bars indicate the standard deviation in Log2-ΔΔCt between the 6 biological replicates. Asterisks (*) indicate significant changes in expression between infected and non-infected mice. The significance level was set at ±2SD of the non-infected group. PGP9.5 (neuronal ubiquitin carboxyl terminal hydrolase), nNOS (neuronal nitric oxide synthase), iNOS (inducible nitric oxide synthase), eNOS (endothelial nitric oxide synthase), TrkA (tropomyosin receptor kinase A), TrkC (tropomyosin receptor kinase C), Th (tyrosine hydroxylase), NGFR, (nerve growth factor receptor), SubP (substance P), VAChT (vesicular acetylcholine transporter), MAP2 (microtubule-associated protein 2).

Of the 11 marker genes investigated, 6 displayed significant differential expression in the heart tissue of *T. cruzi*-infected mice compared with the non-infected controls: nNOS, iNOS, tropomyosin receptor kinase C (TrkC), tyrosine hydroxylase (TH), substance P (SubP), and vesicular acetylcholine transporter (VAChT) (Figure 4). Amongst these, the only gene significantly upregulated was iNOS, which exhibited a 28-fold increase in response to infection, a finding that correlated with anti-iNOS staining of cardiac tissue (Figure 3E). Of the downregulated genes, four of the corresponding proteins have a role in neurotransmission: VAChT (90% reduction), SubP (86% reduction), TH (76% reduction), nNOS (70% reduction). The absence of significant decreases in the pan-neuronal marker protein gene product 9.5 (PGP9.5) or the microtubule-associated protein 2 (MAP2), a structural marker associated with dendritic growth [39], suggested that general infection-induced denervation was not linked with the cardiac abnormalities seen with this infection model, at least during the acute stage. *T. cruzi*-induced iNOS has been shown to contribute to denervation in vitro [40] and enteric neuronal damage in vivo [28].

## 4. Discussion

*T. cruzi* parasites are restricted by immune pressure to a small number of sites in chronically infected mice, predominantly in the GI tract and skin [16,41]. Infection of other tissues and organs tends to be more sporadic and can be influenced by the host:parasite strain combination. For example, ex vivo imaging, which provides an endpoint snapshot of parasite location in tissues and organs, revealed that *T. cruzi* CL Brener parasites can be detected in the hearts of only 10% of BALB/c and C3H/HeN mice during chronic stage infections. In contrast, with strain JR, cardiac infections reach 50% and >80%, respectively [16,18]. In the C3H/HeN model, other tissue sites, such as lung and skeletal muscle, also display similarly high rates of infection, indicating the absence of any intrinsic cardiac tropism. These observations reflect variable frequencies of transient infections in different host–parasite combinations [22]. As a result, it can be inferred that stronger and/or more frequent cardiac immune responses will be induced in the C3H/HeN:JR infection model, resulting in greater collateral damage mediated, for example, by increased peroxynitrite generation [39]. We propose that it is the higher frequency of intermittent cardiac infection in this model that leads to the increased development of disease pathology in a cumulative manner. The particular susceptibility of the heart to progressive chronic disease could result from its low regenerative capacity [42] and the crucial role of neurological homeostasis.

In humans, ECG abnormalities are prevalent in *T. cruzi*-infected individuals, the most common being ventricular (RBBB and LAFB), often progressing to bifascicular blocks. Later manifestations include left ventricular systolic dysfunction, apical aneurysms, high degree atrioventricular block, and sustained and non-sustained ventricular tachycardia [43]. Such cardiac abnormalities can be detected at different stages of infection but are not present in all infected individuals, a profile similar to that observed in the mouse model. A study of asymptomatic *T. cruzi*-seropositive blood donors found a moderate (1.85%) annual increase in the incidence of Chagas cardiomyopathy, although disease was generally mild at the point of diagnosis [44]. Thus, the development of CCC is extremely heterogeneous in both timing and severity, presumably a consequence of diverse host and parasite genetics, complex behavioural and lifestyle issues, and environmental factors.

To further investigate the temporal development of cardiac abnormalities in the C3H/HeN:JR model, we undertook longitudinal heart monitoring. Importantly, this facilitates data acquisition without the requirement of anaesthesia, an intervention that might otherwise perturb cardiac function. Bradycardia, one of the most common clinical presentations in Chagas heart disease, was detected in 33% of strain JR-infected C3H/HeN mice. We observed a significant decrease in HR during the acute phase, which was attributable to the elongation of overlapping PQ, PR, RR, ST, and QT parameters, as depicted in Figure 1. Notably, the prolonged PQ intervals observed in infected mice, in comparison to non-infected age-matched controls, suggest that the infection may be adversely affecting the function of the atrioventricular (AV) node or its associated pathways. This, in turn, could lead to progressive cardiac dysfunction, underscoring the importance of early diagnosis and management of CD. The occurrence of sinus bradycardia (an outcome of sinus node disfunction) and/or ventricular extrasystole (premature ventricular contraction) were also apparent in approximately one third of the ECG profiles obtained from acutely infected mice. As a general trend, these abnormalities became less pronounced when the infection transitioned to the chronic stage (21–25 wpi) and the parasite burden had been reduced by the immune response. ST and QT show abnormalities in ventricular repolarisation, and HRV denotes changes in cardiac autonomic function [45]. The main ECG change reported in *T. cruzi*-infected C57BL/6 mice was an increase in the duration of the PR interval, reflecting a delay in electrical conduction through atrioventricular (AV) nodal conduction in chronically infected mice [46]. No abnormalities in QRS intervals were observed in any mice during the chronic phase of infection, analogous to the situation in humans, where a pooled analysis of 10 studies, comprising >5000 individuals, found no statistically significant difference in prevalence of QRS abnormalities between CD and non-CD patients [9].

Cardiac conductance defects that did develop in the model during the chronic stage were RBBB (24%) and LAFB (9%). In humans, RBBB has been observed in 15% of *T. cruzi*-seropositive individuals, compared with less than 1% of those that were seronegative [47]. In contrast to the JR strain, chronic infection of C3H/HeN mice with the CL Brener strain had a minimal effect on cardiac function, with a slight reduction in HR being the only abnormality identified over the period assessed (Figure 1H). Therefore, the observation that parasite genotype influences the extent of chronic stage fibrosis [16] can be extended to include an impact on cardiac function. Both outcomes can be correlated with differences in the inferred cumulative heart-specific parasite loads during chronic infections with the JR and CL Brener strains. Interestingly, when digestive CD in these models was investigated, severity was greater in C3H/HeN mice than in the BALB/c strain, and JR infections were associated with more severe pathology than those with *T. cruzi* CL Brener [48].

In the C3H/HeN:JR experimental model [18], the extent of cardiac fibrosis is highly variable, ranging from slight to severe, a profile that mirrors the heterogeneous nature of human CCC. Infection of C3H/HeN mice with *T. cruzi* JR results in cardiac inflammation and increased iNOS expression at both the mRNA and protein levels, particularly during the acute phase. This extensive cellular infiltration into cardiac muscle coincides with the suppression of the parasite burden at this stage of the infection (6 wpi) (Figure 3A,B). In this model, mice are also prone to sudden death during this period, with an approximate 10% fatality rate. However, general infection-induced denervation does not appear to be a cause of cardiac disfunction during this stage of the infection, at least based on the expression of transcripts encoding the pan-neuronal marker protein PGP9.5 or MAP2, a structural marker associated with dendritic growth (Figure 4). During chronic stage *T. cruzi* infection, when heart parasitism is considerably reduced (Figure 2C) and not persistent in all mice, there are decreased levels of inflammation and iNOS expression (Figure 3C,G). Interestingly, in this phase of the infection, there is negative association between inflammation and iNOS levels. This could result from disease stage-specific alterations in the inflammatory/anti-inflammatory make-up of the cellular infiltrate, a phenomenon that could influence pathological outcome and merits further study.

Because of the complexity and long-term nature of CD, much of the fundamental research that underpins advances in our understanding of disease pathogenesis and the development of new treatments must be undertaken in experimental models. The ability of these models to capture the spectrum of human disease pathology is critical for translation. As described here, the C3H/HeN:JR host:parasite combination displays many of the diverse features of *T. cruzi*-induced pathology observed in humans and therefore represents a valuable predictive model for experimental research on this debilitating disease. As recently reported [49], ECG in mice can be obtained by telemetry, under anaesthesia, and in conscious animals, depending on the equipment available. It is important to note that in mice, ECG profiles do display some characteristics that are distinct from those of humans, mostly attributable to the HR, which is ~10 times that of humans.

In this exploratory study, we have focussed on a limited number of female mouse:parasite combinations. It will be important to extend this work to capture other variables, such as parasite diversity, together with the gender and genetic background of the host. In addition, studies on cardiac neuronal gene expression in the chronic stage of infection could provide further insights into the mechanisms of electrical disturbances caused by *T. cruzi* infection.

## Figures and Tables

**Table 1:** Primers used for analysis of cardiac neuronal-specific genes. RT-qPCR gene targets and primers. Size refers to the size of the amplicon in base pairs (bp).

Gene	Forward Primer	Reverse Primer	Size (bp)
HPRT1	GCTTGCTGGTGAAAAGGACCTCTCGAAG	CCCTGAAGTACTCATTATAGTCAAGGGCAT	117
PGP9.5	CCTGTGGTACCATCGGGTTG	GGCTCTATCTTCGGGGGACA	125
SubP	CCGACTGGTCCGACAGTGAC	CGCTTGCCCATTAATCCAAA	118
nNOS	ACTGACACCCTGCACCTGAAGA	GTGCGGACATCTTCTGACTTCC	113
iNOS	CAGCTGGGCTGTACAAACCTT	CATTGGAAGTGAAGCGTTTCG	95
eNOS	CCTCGAGTAAAGAATTGGGAAGTG	AACTTCCTTGGAAACACCAGGG	121
TrKA	CGCTGAGTGCTACAACCTTC	GAAAGTCCTGCCGAGCATTC	95
TrKC	TACCTGGCTTCCCAGCACTTTG	GTGTCCTCCCACCCTGTAGTAATC	140
NGFR	GGTGATGGCAACCTCTACAGT	CCTCGTGGGTAAAGGAGTCTA	139
TH	TCCTGCACTCCCTGTCAGAG	CACCGGCTGGTAGGTTTGAT	95
VAChT	CCCTTAAGCGGGCCTTTCATTGAT	AAAGGCAAACATGACTGTGGAGGC	96
MAP2	AGTGGAGGAAGCAGCAAGTGGTGACT	GAGGAGGGAGGATGGAGGAAGGTCT	140
GAPDH	TCCTGCACCACCAACTGCTT	CACGCCACAGCTTTCCAGAG	141

PGP9.5: protein gene product 9.5, SubP: substance P, nNOS: neuronal NOS, iNOS: inducible NOS, eNOS: endothelial NOS, TrKA: tyrosine kinase receptor A, TrkC: tyrosine kinase receptor C, NGFR: nerve growth factor receptor, TH: tyrosine hydroxylase, VAChT: vesicular acetylcholine transporter, MAP2: microtubule-associated protein 2, HRPT1 and GAPDH: internal control genes.

## Data Availability

Data availability under request via corresponding author.

## References

[1] (2023). World Health Organization (WHO). https://www.who.int/news-room/fact-sheets/detail/chagas-disease-(american-trypanosomiasis).

[2] Bonney K.M., Engman D.M. (2008). Chagas heart disease pathogenesis: One mechanism or many?. Curr. Mol. Med..

[3] Pérez-Molina J.A., Molina I. (2018). Chagas disease. Lancet.

[4] Bonney K.M., Luthringer D.J. (2019). Pathology and pathogenesis of Chagas heart disease. Annu. Rev. Pathol..

[5] Pack A.D., Collins M.H. (2018). Highly competent, non-exhausted CD8+ T cells continue to tightly control pathogen load throughout chronic *Trypanosoma cruzi* infection. PLoS Pathog..

[6] Tarleton R.L. (2015). CD8+ T cells in *Trypanosoma cruzi* infection. Semin. Immunopathol..

[7] Malik L.H., Singh G.D. (2015). The Epidemiology, Clinical manifestations, and management of Chagas heart disease. Clin. Cardiol..

[8] Ribeiro A.L., Nunes M.P. (2012). Diagnosis and management of Chagas disease and cardiomyopathy. Nat. Rev. Cardiol..

[9] Rojas L.Z., Glisic M. (2018). Electrocardiographic abnormalities in Chagas disease in the general population: A systematic review and meta-analysis. PLoS Negl. Trop. Dis..

[10] Brito B.O.F., Ribeiro A.L.P. (2018). Electrocardiogram in Chagas disease. Rev. Soc. Bras. Med. Trop..

[11] Di Lorenzo Oliveira C., Nunes M.C.P. (2020). Risk score for predicting 2-Year mortality in patients with Chagas cardiomyopathy from endemic areas: SaMi-Trop Cohort Study. J. Am. Heart Assoc..

[12] Echeverría L.E., Rojas L.Z. (2022). Longitudinal strain by speckle tracking and echocardiographic parameters as predictors of adverse cardiovascular outcomes in chronic Chagas cardiomyopathy. Int. J. Cardiovasc. Imaging..

[13] Salazar-Schettino P.M., Cabrera-Bravo M. (2016). Chagas Disease in Mexico: Report of 14 Cases of Chagasic Cardiomyopathy in Children. Tohoku. J. Exp. Med..

[14] Rodriguez H.O., Guerrero N.A. (2014). *Trypanosoma cruzi* strains cause different myocarditis patterns in infected mice. Acta Trop..

[15] Queiroga T.B.D., Pereira N.S. (2021). Virulence of *Trypanosoma cruzi* strains is related to the differential expression of innate immune receptors in the heart. Front Cell Infect. Microbiol..

[16] Lewis M.D., Francisco A.F. (2016). Host and parasite genetics shape a link between *Trypanosoma cruzi* infection dynamics and chronic cardiomyopathy. Cell Microbiol..

[17] Caldas I.S., Diniz L.F. (2017). Host genetics background influence in the intragastric *Trypanosoma cruzi* infection. Acta Trop..

[18] Francisco A.F., Jayawardhana S. (2018). Assessing the effectiveness of curative benznidazole treatment in preventing chronic cardiac pathology in experimental models of Chagas disease. Antimicrob. Agents Chemother..

[19] Tarleton R.L., Zhang L. (1997). “Autoimmune rejection” of neonatal heart transplants in experimental Chagas disease is a parasite-specific response to infected host tissue. Proc. Natl. Acad. Sci. USA.

[20] Bonney K.M., Engman D.M. (2015). Autoimmune pathogenesis of Chagas heart disease: Looking back, looking ahead. Amer. J. Pathol..

[21] Lewis M.D., Francisco A.F. (2014). Bioluminescence imaging of chronic *Trypanosoma cruzi* infections reveals tissue-specific parasite dynamics and heart disease in the absence of locally persistent infection. Cell Microbiol..

[22] Lewis M.D., Kelly J.M. (2016). Putting *Trypanosoma cruzi* dynamics at the heart of Chagas disease. Trends. Parasitol..

[23] Lewis M.D., Fortes Francisco A. (2015). A new experimental model for assessing drug efficacy against *Trypanosoma cruzi* infection based on highly sensitive in vivo imaging. J. Biomolec. Screen..

[24] Branchini B.R., Ablamsky D.M. (2010). Red-emitting luciferases for bioluminescence reporter and imaging applications. Anal. Biochem..

[25] Schuldt A.J.T., Hampton T.J. (2004). Electrocardiographic and other cardiac anomalies in beta-glucuronidase-null mice corrected by nonablative neonatal marrow transplantation. Proc. Natl. Acad. Sci. USA.

[26] Mabe A.M., Hoover D.B. (2009). Structural and functional cardiac cholinergic deficits in adult neurturin knockout mice. Cardiovasc. Res..

[27] Xing S., Tsaih S.W. (2009). Genetic influence on electrocardiogram time intervals and heart rate in aging mice. Am. J. Physiol. Heart Circ. Physiol..

[28] Arantes R.M.E., Marche H.H.F. (2004). Interferon-gamma-induced nitric oxide causes intrinsic intestinal denervation in *Trypanosoma cruzi*-infected mice. Am. J. Pathol..

[29] Livak K.J., Schmittgen T.D. (2001). Analysis of relative gene expression data using real-time quantitative PCR and the 2(-Delta Delta C(T)) method. Methods.

[30] Eickhoff C.S., Lawrence C.T. (2010). ECG detection of murine chagasic cardiomyopathy. J. Parasitol..

[31] Roman-Campos D., Sales-Junior P. (2013). Novel insights into the development of chagasic cardiomyopathy: Role of PI3Kinase/NO axis. Int. J. Cardiol..

[32] Navarro I.C., Ferreira F.M. (2015). MicroRNA transcriptome profiling in heart of *Trypanosoma cruzi*-infected mice: Parasitological and cardiological outcomes. PLoS Negl. Trop. Dis..

[33] Vilar-Pereira G., Carneiro V.C. (2016). Resveratrol reverses functional Chagas heart disease in mice. PLoS Pathog..

[34] Carbajosa S., Rodríguez-Angulo H.O. (2018). L-arginine supplementation reduces mortality and improves disease outcome in mice infected with *Trypanosoma cruzi*. PLoS Negl. Trop. Dis..

[35] Santos-Miranda A., Joviano-Santos J.V. (2020). Reactive oxygen species and nitric oxide imbalances lead to in vivo and in vitro arrhythmogenic phenotype in acute phase of experimental Chagas disease. PLoS Pathog..

[36] Chuenkova M.V., Pereiraperrin M. (2011). Neurodegeneration and neuroregeneration in Chagas disease. Adv. Parasitol..

[37] Zhang Y.H., Jin C.Z. (2014). Molecular mechanisms of neuronal nitric oxide synthase in cardiac function and pathophysiology. J. Physiol..

[38] Burger D.E., Lu X. (2009). Neuronal nitric oxide synthase protects against myocardial infarction-induced ventricular arrhythmia and mortality in mice. Circulation.

[39] Harada A., Teng J. (2002). MAP2 is required for dendrite elongation, PKA anchoring in dendrites, and proper PKA signal transduction. J. Cell Biol..

[40] Almeida-Leite C.M., Galvão L.M. (2007). Interferon-gamma induced nitric oxide mediates in vitro neuronal damage by *Trypanosoma cruzi*-infected macrophages. Neurobiol. Dis..

[41] Ward A.I., Lewis M.D. (2020). In vivo analysis of *Trypanosoma cruzi* persistence foci at single-cell resolution. mBio.

[42] Porrello E.R., Mahmoud A.I. (2011). Transient regenerative potential of the neonatal mouse heart. Science.

[43] Rassi A., Rassi A. (2000). Chagas’ heart disease. Clin. Cardiol..

[44] Sabino E.C., Ribeiro A.L. (2013). Ten-year incidence of Chagas cardiomyopathy among asymptomatic *Trypanosoma cruzi*-seropositive former blood donors. Circulation.

[45] Hevia-Montiel N., Perez-Gonzalez J. (2022). Machine Learning-Based Feature Selection and Classification for the Experimental Diagnosis of *Trypanosoma* cruzi. Electronics.

[46] Tucci A.R., de Oliveira F.O.R. (2020). Role of FAK signaling in chagasic cardiac hypertrophy. Braz. J. Infect. Dis..

[47] Williams-Blangero S., Magalhaes T. (2007). Electrocardiographic characteristics in a population with high rates of seropositivity for *Trypanosoma cruzi* infection. Am. J. Trop. Med. Hyg..

[48] Khan A.A., Langston H.C. (2021). Local association of *Trypanosoma cruzi* chronic infection foci and enteric neuropathic lesions at the tissue micro-domain scale. PLoS Pathogens..

[49] Haro P., Hevia-Montiel N. (2023). ECG marker evaluation for the machine-learning-based classification of acute and chronic phases of *Trypanosoma cruzi* infection in a murine model. Trop. Med. Infect. Dis..

